# Effects of COVID-19 nursing home restrictions on people with dementia involved in a Supportive Care programme

**DOI:** 10.3389/frhs.2024.1440080

**Published:** 2024-09-17

**Authors:** Omar Paccagnella, Francesco Miele, Angelica Guzzon, Federico Neresini, Vincenzo Rebba, Michela Rigon, Giovanni Boniolo

**Affiliations:** ^1^ Department of Statistical Sciences, University of Padova, Padua, Italy; ^2^ Department of Political and Social Sciences, University of Trieste, Trieste, Italy; ^3^ Department of Economics, Ca’ Foscari University of Venice, Venice, Italy; ^4^ Department of Philosophy, Sociology, Education and Applied Psychology, University of Padova, Padua, Italy; ^5^ Department of Economics and Management “Marco Fanno”, University of Padova, Padua, Italy; ^6^ CRIEP—Interuniversity Research Centre of Public Economics, Italy; ^7^ Fondazione OIC Onlus, Padua, Italy; ^8^ Department of Neuroscience and Rehabilitation, University of Ferrara, Ferrara, Italy

**Keywords:** COVID-19, dementia, neuropsychiatric inventory, nursing homes, Supportive Care

## Abstract

**Background:**

Supportive Care is a person-centred approach encompassing non-pharmacological interventions targeted towards persons with dementia to contain the effects of their behavioural disorders, improving their quality of life.

**Aims:**

To investigate the effects of lockdown restrictions during the first wave of COVID-19 pandemic on behavioural symptoms of patients involved in a Supportive Care programme in an Italian nursing home.

**Methods:**

Analysis is based on Neuropsychiatric Inventory (NPI) scores and related symptoms data collected before (October/November 2019) and after (July 2020) the introduction of COVID-19 restrictions on a non-random sample of 75 patients living in two units of the facility: 38 involved in a Supportive Care programme and 37 receiving standard care (Control). Group performances were compared over time according to univariate statistics and Latent Class Analysis (LCA).

**Results:**

NPI scores and number of reported symptoms in NPI evaluations increased over time among Supportive Care patients with dementia and decreased in the Control group. Differences are statistically significant. LCA resulted in 3-classes and 5-classes specifications in the two time-occasions.

**Discussion:**

Supportive Care patients showed a worsening in behavioural and psychological symptoms after the first pandemic wave, as opposed to the elderly not involved in the programme. LCA showed that patients in the two groups differed according to the combinations of NPI symptoms.

**Conclusions:**

The discontinuation of a Supportive Care programme due to COVID-19 restrictions had strong negative effects on nursing home persons with dementia involved in the programme: Supportive Care interventions are important in controlling the psycho-behavioural symptoms associated with dementia.

## 1 Introduction

The World Health Organization has recently described dementia as “an umbrella term for several diseases that are mostly progressive and affect memory, other cognitive abilities and behaviour” (1). For many years, dementia care has been based on the so called *standard medical approach* (2) and mainly treated with drugs (i.e., anti-anxiety, antidepressant and anti-psychotic medications) (3, 4).

Recently, such over-treatment with drugs to manage the physiological and behavioural symptoms of dementia has been criticised, because of negative effects at the individual (5) and collective (6, 7) level. Yet, occurrence of behavioural symptoms (such as depression, anxiety, agitation, and so on) are also related to the way the caregivers treat the individual suffering from dementia (8–10). To this aim, a person-centred care has been proposed as an alternative (11–14): dementia varies from person to person and does not progress linearly, therefore individual needs and interpersonal relationships are the milestones for building good dementia care.

Starting from these principles, the concept of Supportive Care (SC) has been introduced, meaning as “a full mixture of biomedical dementia care, with good quality, person-centred, psychosocial, and spiritual care under the umbrella of holistic palliative care throughout the course of the person's experience of dementia, from diagnosis until death” (15). Born in the geriatric context, the Supportive Care approach has been now discussed in the oncological literature (16). Focusing on the key interventions (person-centred, palliative and multi-disciplinary care) in supporting people with dementia, an in-depth literature review (17) highlighted the positive and the critical implications of the implementation of Supportive Care Interventions (SCI) for long-term care organisations. There is evidence that the burden to caregivers of patients living with dementia also decreased significantly (18).

During spring 2020, almost all countries in the world experienced lockdown and home confinement because of the first wave of COVID-19 pandemic caused by the SARS-CoV-2 coronavirus. These measures strongly affected social and cognitive dimensions, worsening neuropsychiatric traits and neurobehavioral manifestations of older people, particularly in vulnerable patients such as people with dementia (19–23).

In this work we aim to produce progress in studies on dementia investigating the effects of the first lockdown during the COVID-19 outbreak on the behavioural symptoms of dementia of elderly people involved in a SCI project in an Italian long-term care facility. More specifically, our goal is to study whether older people suffering from dementia and living in a facility organised to fulfil SCI worsened their psycho-behavioural disorders after the first wave of the COVID-19 outbreak, because of the interruption of some care practices/activities planned by the project (with the aim of limiting the interactions among residents and, consequently, the risk of infection). Therefore, the purpose of this paper is not to assess the impact of the disruption of this SC program, but to investigate the effects of lockdown measures in a group of patients with dementia (PwD) involved in SC activities. In addition, this analysis may offer useful lessons for improving the quality of life of dementia patients in case of future pandemics or similar events, suggesting the need of not completely deactivating SC programmes, but maintaining those components characterised by lower risks of contagion.

## 2 Materials and methods

The present study considers a sample of 75 individuals, equally divided between 38 patients treated with SCI and 37 belonging to a Control group. They are a subsample of patients involved in a Supportive Care project (funded by the *Fondazione OIC Onlus*), that started in 2014 and was discontinued in March 2020 as a consequence of the measures adopted by the Italian authorities to prevent the spread of the COVID-19 outbreak.

### 2.1 The original Supportive Care project

This paper is based on data partly collected within a project (started in 2014) focused on Supportive Care for elderly people with severe cognitive impairment developed at the *Fondazione OIC Onlus* (Padua, Italy). The original project named “Nuovi Passi” (24) was aimed at evaluating the effects of a Supportive Care programme on care processes, organisational cultures, and quality of life in some long-term care units for older people. Its main outcome was to improve care practices and organisational contexts in which PwD could benefit from motor, sensorial, artistic and relational activities of various kinds provided alongside conventional medical care. The goal of this type of programme was to improve the behavioural and psychological symptoms related to dementia, rather than the cognitive abilities of the patients. This programme also aimed to improve the organisational processes and the quality of working-life of the PwD caregivers. In the original project development, 134 patients with moderate/severe cognitive decline and severe behavioural disorders were selected on the basis of both the Short Portable Mental Status Questionnaire (SPMSQ) and a summary evaluation of behavioural symptoms^1^. Three different units of the *Fondazione OIC Onlus* were involved in the study, in order to create a SCI-treated and a Control group of patients, having similar size in each. The original project planned to evaluate patients and staff members at some scheduled temporal periods. In October/November 2019 (called T0 for this paper), a first set of information concerning the patients of this project was collected. The COVID-19 outbreak started before the collection of follow-up information, originally planned in April/May 2020 (six months after T0).

### 2.2 Participants in the study

In July 2020 (hereafter called T1), neuropsychiatric inventory scores were collected in a (non-random) subsample of the patients living in two of the units originally involved in the project^2^. No specific inclusion/exclusion criteria, additional to those used to select the patients considered in the original project (specified in Section 2.1) were adopted to select the individuals: all patients living in these two units of the facility at T1 were evaluated. However, for those who were involved in the original Supportive Care project, the NPI evaluations at T1 were linked with some information available from the data collected at T0. Therefore, the final sample of this work is composed of 75 individuals: 38 treated and 37 untreated. The year of admission ranges from 2014 to 2019 for the SCI patients and from 2012 to 2019 for the Control group.

### 2.3 Measures

Neuropsychiatric Inventory (NPI) is a valid and reliable tool to evaluate behavioural and psychological symptoms in patients with dementia, which is the main goal of the Supportive Care proposal (25, 26). The available information includes the total score collected for each patient at T0 and T1, as well as its disaggregation by symptoms. For each NPI symptom, an individual dummy variable is created both at T0 and T1, equal to 1 if the symptom reported a non-zero score and 0 otherwise. Cognitive impairments are evaluated by the Mini-Mental State Examination (MMSE) (27) at T0 and the Short Portable Mental Status Questionnaire (SPMSQ) (28) at the time of admission to the nursing home. Barthel index (29) is helpful to measure functional disability, i.e., the degree of assistance required by the patient on mobility and self-care ADL items (in this case, it was assessed at the admission to the facility). Data were collected according to the validated Italian versions of the assessment tools.

### 2.4 Statistical analysis

Values are reported as mean ± standard deviation (sd). To estimate within group differences (across the two time points), separately for each group, the Wilcoxon Signed Rank test was used. To estimate group differences at the same time point, the Wilcoxon Mann-Whitney test and test for proportion were used, according to the nature of the variable.

The Latent Class Analysis (LCA) is a powerful way to classify individuals in latent (unobserved) subgroups when categorical indicators are studied (30, 31). More specifically, this approach allows to identify meaningful cluster of profiles depending on the different combinations of reported items that compose the NPI evaluation. LCA is developed sequentially from a 2 to 6-class model. The final model is chosen according to information criteria (BIC, AIC, etc.), classification statistics (Classification Errors, Entropy R^2^ and Standard R^2^), size and interpretability of the latent classes, as well as Bivariate Residuals (BVR) estimates. The *local independence assumption* (32) is a key hypothesis in any LC analysis and BVRs are a way to detect its violation [a value of 3.84 for each pair is suggested as cut-off (33)]. LCA was performed using LatentGOLD 5.0 (34), while all other analyses were performed using STATA15.

## 3 Results

Main characteristics of the sample are reported in Table 1. It was mostly composed of women, with an average age greater than 85 years at T0 (a little bit higher in the Control group than in the SCI one, even if not statistically different). According to the MMSE at T0, about half of the patients in the Control group suffered from a moderate cognitive decline, while in the SCI group the proportion of not-administrated MMSE was particularly large. This is confirmed by the SPMSQ scores evaluated at the time of admission: on average, this group of SCI patients showed stronger cognitive impairments than the other group. On the other hand, according to the NPI scoring at T0, neuropsychiatric symptom profiles for the Control group patients are significantly worse than the SCI patients. Since the two groups were not created by randomization, they showed some differences in cognitive and functional characteristics before the start of the pandemic. However, in the period between the two evaluations (about 9/10 months), the COVID-19 outbreak and the lockdown measures were the most relevant events experienced by all patients.

**Table 1:** Main characteristics of the analytical sample (*n* = 75).

	SCI group	Control group	Test
At T0			
Female (*n*,%)	28 (73.7%)	35 (94.6%)	−2.470 (*p* = 0.014)
Age (mean, sd)	85.05 (7.13)	86.49 (7.35)	−1.046 (*p* = 0.299)
NPI (mean, sd)	16.97 (10.53)	54.16 (18.03)	−6.848 (*p* = 0.000)
MMSE (*n*,%)			
N.A.	13 (34.2%)	2 (5.4%)	
< 10	20 (52.6%)	17 (45.9%)	
10–19	5 (13.2%)	18 (48.7%)	
At the admission in nursing home			
SPMSQ (mean, sd)	9.61 (0.790)	8.38 (2.166)	3.143 (*p* = 0.002)
Barthel index (mean, sd)	61.50 (26.51)	91.69 (15.68)	−5.187 (*p* = 0.000)

Test of proportion for gender and the Wilcoxon Mann-Whitney for age, NPI, SPMSQ and Barthel index were used. No statistical test for MMSE was performed because of the large number of “N.A.” values in the SCI group.

Table 2 compares the differences over time in NPI results, by unit. While we observed a deterioration of the psycho-behavioural disturbances among the SCI patients (both according to the total NPI score and the number of reported NPI symptoms), the opposite result occurred in the Control unit: all comparisons were statistically significant at a probability level α = 0.01.

**Table 2:** Comparison of the NPI results over time, by unit.

	SCI group			Control group		
	T0	T1	Wilcoxon test	T0	T1	Wilcoxon test
Score (mean, sd)	16.97 (10.53)	23.36 (12.00)	–3.309 (*p* = 0.001)	54.16 (18.03)	44.97 (17.86)	2.745 (*p* = 0.005)
Number of reported symptoms (mean, sd)	3.45 (2.14)	5.03 (2.39)	–3.763 (*p* = 0.000)	8.95 (1.96)	7.95 (2.27)	3.288 (*p* = 0.001)

Table 3 reports the average proportion of each NPI symptom in its total score value, by time-occasion and unit. Consistently with Table 2 results, the number of reported items was larger in the Control group than SCI in both periods, even if from T0 to T1 we observed an increase for all proportions of the SCI group and a reduction for almost all percentages of the other unit.

**Table 3:** Proportions of the reported NPI symptoms at T0 and T1, by unit.

Symptom	SCI group		Control group	
	T0	T1	T0	T1
Delusions	42.1%	63.2%	94.6%	67.6%
Hallucinations	15.8%	47.4%	78.4%	59.5%
Agitation/aggression	18.4%	52.6%	86.5%	83.8%
Depression/dysphoria	34.2%	44.7%	83.4%	81.1%
Anxiety	36.8%	44.7%	94.6%	94.6%
Elation/euphoria	7.9%	10.5%	8.1%	18.9%
Apathy/indifference	50.0%	65.8%	83.8%	75.7%
Disinhibition	7.9%	13.2%	32.4%	27.0%
Irritability/lability	36.8%	44.7%	83.8%	86.5%
Motor disturbance	47.4%	50.0%	89.2%	75.7%
Nigh-time behaviours	21.1%	28.9%	78.4%	62.2%
Appetite/eating	26.3%	36.8%	81.1%	62.2%

We then performed LCA on NPI symptom dummies, separately for the two time-occasions. Two- to six-class models were considered: according to multiple fit indices (see Supplementary Table A1 in the Supplementary Materials), we chose a three-class and a five-class specification for T0 and T1, respectively.

According to the individual probability (conditional of belonging to the LC) of reporting each NPI item (see Supplementary Table A2 in the Supplementary Materials), Table 4 labels each cluster, by time: LC1 was characterised by patients with high probability of reporting a *very large number of symptoms* (the combinations include almost all items, except for elation and disinhibition); on the contrary, LC3 was characterised by people with a low probability to have any items, except for apathy (this means that the NPI score depends on the evaluation of apathy only, or on a very limited number of symptoms, one of which is usually apathy). LC2 was in between the previous clusters, because it collected individuals having high probability for a limited number of symptoms (i.e., *less than half*) and apathy did not play any role. These three clusters were replicated at T1. More precisely, moving from T0 to T1: the *very large number of reported symptoms* class did not change its characterisation, but reduced its size; the *less than half of reported symptoms* class reduced its size and showed a more important role of the delusions and agitation/aggression items at T1; the *very few symptoms—usually apathy is one of them* cluster did not change its characterisation, but increased its size. In addition, the other two classes at T1 were LC4 (characterised by patients with high probability of reporting *about half of symptoms*) and LC5 (elderly with *large number of reported symptoms—more than half*, where the elation and disinhibition components now played a role). Changes between classes over time are summarised in Supplementary Figures A1, A2 and Table A3 (reporting the number of individuals underlying these graphs) of the Supplementary Materials.

**Table 4:** Latent class analysis on NPI symptoms: general description and size of the classes at T0 and T1, by unit.

Latent class	Description	SCI group	Control group	Total
T0				
1	Very large number of reported symptoms	3	33	36 (48.0%)
2	Less than half of reported symptoms	19	2	21 (28.0%)
3	Very few symptoms—usually apathy is one of them	16	2	18 (24.0%)
Total		38	37	75 (100.0%)
T1				
1	Very large number of reported symptoms	7	17	24 (32.0%)
2	Less than half of reported symptoms	6	3	9 (12.0%)
3	Very few symptoms—usually apathy is one of them	18	3	21 (28.0%)
4	About half of reported symptoms	3	8	11 (14.7%)
5	Large number of reported symptoms—more than half	4	6	10 (13.3%)
Total		38	37	75 (100.0%)

Table 4 also shows the size of each cluster, by unit. Before the COVID-19 outbreak, almost all PwD of the Control group belonged to the first class, while SCI patients were about equally divided among clusters 2 and 3. After the first wave of the outbreak, this partition between units was no longer valid: for each unit, about half of the patients belonged to a specific LC (that is, *very few symptoms—usually apathy is one of them* for the SCI group and *very large number of reported symptoms* for the Control unit), while the remaining were classified in all other classes. At T1, the *about half of reported symptoms* class was largely composed of patients belonging to the Control group, while in the *less than half of reported symptoms* cluster there was a prevalence of SCI individuals.

## 4 Discussion

The main finding of this study is that older people living in a facility organised to fulfil SCI worsened their psycho-behavioural disturbances after the first wave of the COVID-19 pandemic, while an improvement was seen among patients not involved in these supportive practices. The discontinuation of a SC programme due to the Italian COVID-19 restrictions had strong negative effects on nursing home residents involved in this programme in terms of neuropsychiatric symptoms triggered by the protracted isolation. Indeed, some care practices/activities planned by the SC project were no longer regularly implemented to the PwD. In particular, two key interventions of SC models were suspended or dramatically reduced during the first wave of the COVID-19 pandemic: (i) socio-educational activities, which are aimed at general enhancement of memory, sociality, spirituality and cognitive and motor skills of people with dementia; (ii) interdisciplinary meetings, that are aimed at coordinating professionals with different backgrounds (psychologists, educators, nurses, physiotherapists and care aides). On the other hand, the Control unit was more isolated from the outside world during that lockdown, and this in turn helped to reduce some sources of stress for the unit staff [as shown by the literature, interactions with relatives can be a source of conflict for the staff—see for instance (35)] and the Control group patients. It is reasonable to think that also SCI patients benefitted from a similar stress reduction due to the imposed isolation, but this was not able to balance the strong negative consequences of the lack of the interventions provided by the Supportive Care programme. To support this hypothesis, we note (as will be discussed later) that also some SCI patients showed an improvement of their NPI results, but the majority of them had however a strong deterioration.

Since SCI and Control group patients lived in two independent units, it could be interesting to investigate the effects due to other organisational differences in these structures. Indeed, as shown by Table 4, before the COVID-19 outbreak the results of the LCA based on the NPI evaluations showed a unit-specific differentiation of the PwD: one class was almost all composed of the elderly from one unit, while two other classes almost equally divided those living in the other unit. After the COVID-19 outbreak, this classification changed and may explain the findings in Table 3. The Control group showed much more stability over time on group belonging than the SCI one. As highlighted by Supplementary Figure A2 in the Supplementary Materials, from T0 to T1 Control group patients either remained in the same LC or moved to a healthier class (that is, characterised by a lower number of reported NPI symptoms with respect to the LC in T0). This was not true for the SCI group, where for instance patients in the “*less than half of reported symptoms*” class at T0 were assigned to each of the five classes at T1 (Supplementary Figure A1 in the Supplementary Materials); however, also in the SCI unit few individuals moved to a class characterised by a lower number of reported NPI symptoms with respect to the class belonging at T0.

Although the average number of reported symptoms in the NPI score was much larger in the Control group than in the SCI one, the patients in the two units differed also according to the combination of NPI symptoms displayed. Neuropsychiatric symptoms occurred in the majority of PwD over the course of the disease: apathy, depression and agitation were the most important, and, to a lesser extent, sleep and eating disturbances (36, 37). According to Table 4, in the SCI group apathy was the most reported symptom, both at T0 and at T1, but not in the Control group, where anxiety and delusions were the most reported items at T0, and anxiety and irritability at T1. Among SCI patients, from T0 to T1 we observed an increase of the reported proportions for all NPI symptoms, with agitation and hallucinations showing particularly large variations. On the other hand, in the Control group, all symptoms reduced their percentages between T0 and T1, except for elation, irritability (an increase in the period) and anxiety (no change); the largest negative variation was for delusions. The profiles of the psycho-behavioural disturbances that characterised the elderly in the Control unit were different from the ones in the SCI group.

Unfortunately, we had limited information on the social and health characteristics of the individuals living in the two units. They were similar according to age and (to some extent) gender composition, but not with respect to cognitive functioning: the Control group showed a proportion of patients suffering from a moderate cognitive decline larger than the SCI group (where, at the same time, the proportion of not administrated MMSE tests was unexpectedly large). This finding was particularly interesting considering that the severity of neuropsychiatric symptoms assessed by NPI scores was greater in the Control group than in the SCI. Although the usefulness of the MMSE in the identification of patients with mild cognitive impairment or dementia is limited (38, 39), there is evidence in the literature that patients diagnosed with mild cognitive impairment can also display neuropsychiatric symptoms (40), but the strength of this relationship varies considerably according to the subgroups obtained on the basis of the correlated psychological symptoms that can be identified (41, 42).

Moreover, such symptoms may lead to different trajectories to developing dementia, but conflicting results have been found. Irritability and apathy appeared to be the determining factors in developing dementia by (43), while apathy and depression have been shown to be the most predictive of progression by (44). (45) found evidence that an increase in neuropsychiatric symptoms burden (through NPI scores) over time predicts conversion to Alzheimer disease, while a non-conversion was favored by a stability of symptoms. The conditional probabilities of reporting symptoms in our LC analysis (Supplementary Table A2 in the Supplementary Materials) showed that apathy played an important role in identifying the classes, as well as anxiety and irritability, in both time occasions. At T1, the agitation and (to a lesser extent) depression items became more influential in profiling PwD.

It was also worth noting that at T0 all SCI patients were involved in the project, but with different time spans (patients admitted close to 2014 may have benefitted from more care practices/activities of the project compared to those enrolled later on). However, we observed that the larger the time span, the lower the NPI score. Supplementary Table A4 in the Supplementary Materials reports the linear correlation between time span in the nursing home (i.e., years of being in the nursing home at T0) and the changes from T0 to T1 in both NPI score and the number of NPI reported items. This relationship was positive and statistically significant for SCI patients, while it was close to 0 and not statistically significant for the other group: patients who benefitted from the SC practices for a longer time revealed the largest NPI deterioration after stopping these activities.

### 4.1 Strengths and limitations of the analysis

Our study provided important insights about the negative effects of lockdown measures during the first wave of the COVID-19 pandemic on behavioural symptoms of patients suffering from dementia involved in an innovative Supportive Care programme implemented in an Italian nursing home.

A number of limitations in this study should be acknowledged. The two groups were not created by a random assignment and their sample size is rather low. Therefore, we cannot ascertain causal relationships of the COVID-19 outbreak on the SCI patients. We could use the difference-in-differences solution to estimate the impact of the discontinuation of the SC programme due to the first wave COVID-19 restrictions in Italy. However, we did not have additional information to assess the validity of the underlying assumption of equal trends over time evaluating the outcome changes in the SC and Control groups (46). Thus, some time-dependent LCAs (i.e., Markov LCA) cannot be performed, even if data were longitudinally collected. Moreover, information on the PwD characteristics was very limited (we had partial access to individual information collected in T0) and the lack of individual socio-economic variables did not allow to perform in-depth analyses. Therefore, results cannot be generalised to the Italian long-term care facility environment.

## 5 Conclusions

This study provided a first evidence of the effects of the first lockdown during COVID-19 outbreak on the behavioural conditions of older people suffering from dementia in a long-term care facility in Italy. SCI patients showed a significant deterioration of these conditions in a short period (less than one year between the two measurements), as opposed to the older people who were not involved in the “Supportive Care for elderly people with severe cognitive impairment” project. These findings confirmed that SCI (interrupted during the COVID-19 pandemic by the forced lockdown) were important in controlling the behavioural and psychological symptoms associated with dementia. Our results also suggested that the assessment of neuropsychiatric symptoms (through latent class analyses able to identify substantial and meaningful subgroups of these profiles among the patients) may provide invaluable information to establish specific treatment strategies and develop customised interventions aimed at slowing down the progression to dementia in these pandemic and long-term care facility contexts, mitigating the higher risk of negative psychosocial effects.

Finally, it was possible to draw useful lessons from the analysed situation of interruption, in order to improve life quality of PwD in case similar events occur in the future, by maintaining at least those cognitive stimulation components of the SC programme (i.e., music therapy) which may be characterised by lower risks of infection.

## Data Availability

Data has been obtained from a third party. The data analysed in this study was obtained from Fondazione OIC Onlus (Padova, Italy). The following restrictions apply: data access is restricted to protect patient confidentiality and participant privacy, and to protect proprietary information. The raw data will be made available for a replicability check of the findings of this article upon request with permission of the third party and after a declaration of confidentiality. Requests to access this dataset should be directed to the corresponding author.

## Footnotes

^1^The assessment of the cognitive decline (according to SPMSQ) and behavioural symptoms are based on some indicators adopted by the Veneto Region of Italy (to which Padua belongs administratively) for the multidimensional evaluation of the non-autonomous older people and collected at the time of the admission of the patient within the nursing home.

^2^Within each unit, NPI evaluations were collected by the same psychologists of the *OIC* staff in both occasions.
