# Supplementary material for: Effects of COVID-19 nursing home restrictions on people with dementia involved in a Supportive Care programme
Source: Front Health Serv. 2024 Sep 17;4:1440080. doi: 10.3389/frhs.2024.1440080 (PMC11447520; doi:10.3389/frhs.2024.1440080)

SUPPLEMENTARY MATERIALS

Table A1 LCA on NPI symptoms: model fit indices

# LCs	AIC	BIC	Entropy R^2	Standard R^2	# large BVRs	Classification errors	# small size LCs (<5%)
				**T0**			
2	217.88	102.00	0.941	0.952	3	0.0169	0
3	209.38	123.63	0.965	0.975	0	0.0087	0
4	210.11	154.49	0.930	0.935	1	0.0273	0
5	212.14	186.65	0.937	0.937	1	0.0272	0
6	Cannot be estimated (negative degrees of freedom)						
				**T1**			
2	300.80	184.93	0.826	0.857	5	0.0460	0
3	298.03	212.28	0.810	0.828	3	0.0698	0
4	292.93	237.31	0.876	0.881	0	0.0516	0
5	287.35	261.86	0.922	0.926	0	0.0323	0
6	Cannot be estimated (negative degrees of freedom)						

Table A2 LCA on NPI symptoms: individual clusters and conditional probabilities of reporting symptoms at T0 and T1

**Symptoms**	**T0**			**T1**				
	**1**	**2**	**3**	**1**	**2**	**3**	**4**	**5**
LC size (per cent)	48.0	28.0	24.0	32.0	28.0	14.7	13.3	12.0
Delusions	0.914	0.481	0.450	0.969	0.303	0.191	0.792	0.989
Hallucinations	0.833	0.151	0.112	0.863	0.425	0.205	0.802	0.016
Agitation/Aggression	0.915	0.289	0.010	0.836	0.208	0.994	0.994	0.665
Depression/Dysphoria	0.911	0.150	0.455	0.825	0.480	0.905	0.623	0.122
Anxiety	0.967	0.570	0.132	0.952	0.205	0.755	0.794	0.992
Elation/Euphoria	0.111	0.048	0.056	0.087	0.001	0.003	0.880	0.003
Apathy/Indifference	0.886	0.018	0.989	0.820	0.858	0.993	0.423	0.017
Disinhibition	0.361	0.050	0.058	0.181	0.053	0.094	0.748	0.114
Irritability/Lability	0.891	0.526	0.112	0.802	0.110	0.993	0.993	0.771
Motor disturbance	0.939	0.619	0.240	0.997	0.208	0.370	0.794	0.772
Nigh-time behaviours	0.829	0.198	0.176	0.789	0.159	0.460	0.481	0.235
Appetite/Eating	0.830	0.291	0.231	0.786	0.343	0.623	0.222	0.227

Table A3 LCA on NPI symptoms: changes in cluster belonging from T0 to T1 by facility, n=75

**Cluster – T0**	**Cluster – T1**					
	1	2	3	4	5	Total
			**SCI group**			
1	2	0	1	0	0	3
2	4	5	1	3	6	19
3	1	13	1	1	0	16
Total	7	18	3	4	6	38
			**Control group**			
1	17	1	8	6	1	33
2	0	0	0	0	2	2
3	0	2	0	0	0	2
Total	17	3	8	6	3	37

Table A4 Linear correlation between the years of being in nursing home at T1 and the changes in NPI evaluations from T0 to T1

Correlation between	**SCI group**	**Control group**
Change in NPI score and timespan in nursing home	0.355 (*p*=0.029)	– 0.053 (*p*=0.757)
Change in number of NPI reported symptoms and timespan in nursing home	0.346 (*p*=0.033)	– 0.052 (*p*=0.759)

Figure A1 LCA on NPI symptoms: changes in cluster belonging between T0 and T1, SCI group

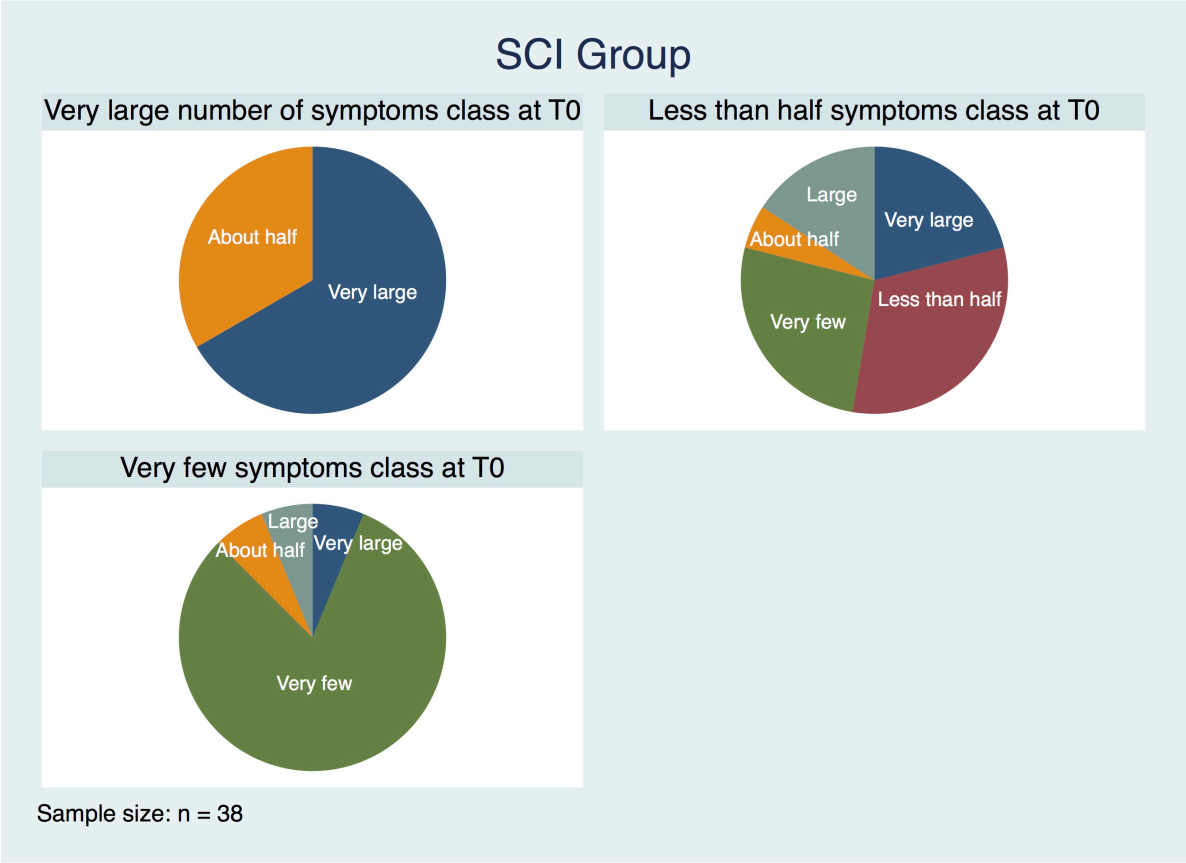

Figure A2 LCA on NPI symptoms – changes in cluster belonging between T0 and T1, Control group

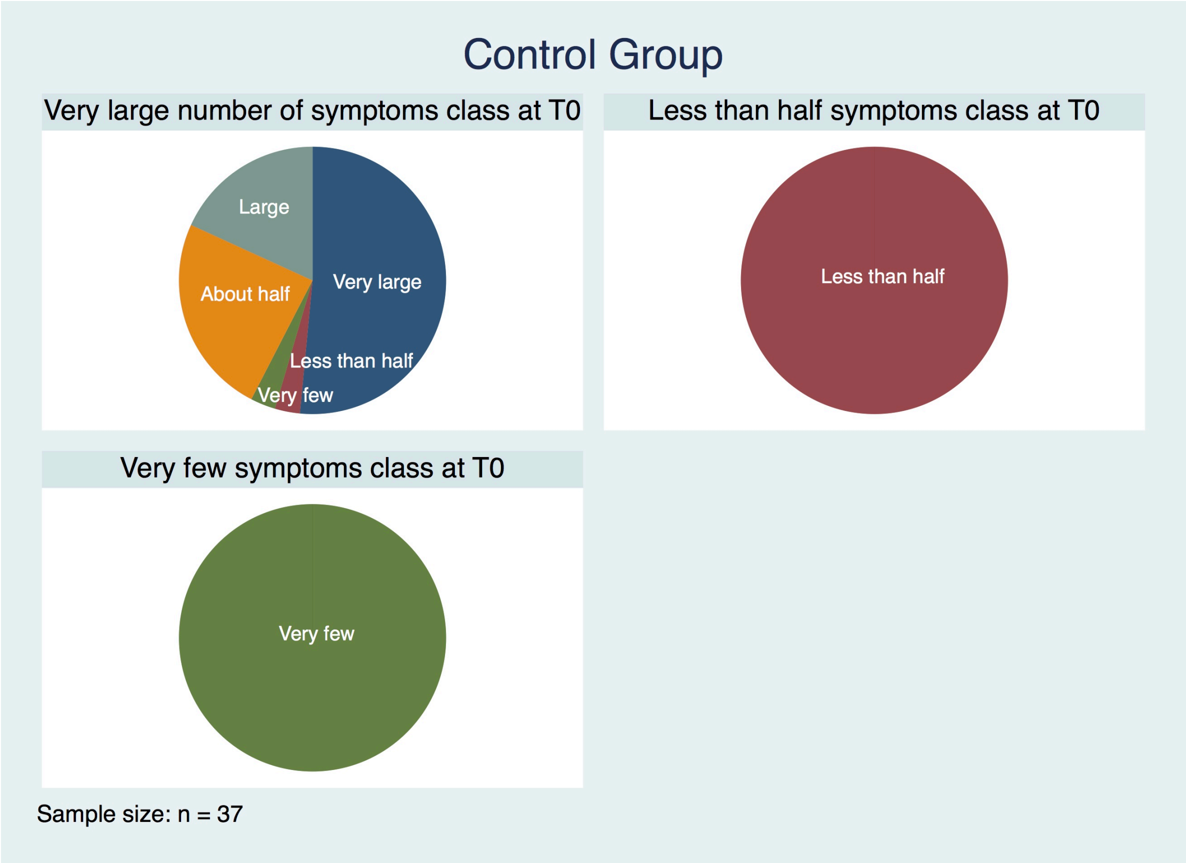

Supplement: Supplementary file 1 [file Datasheet1.pdf]
